# In Vitro Comparison of Three Intraoral Scanners for Implant—Supported Dental Prostheses

**DOI:** 10.3390/dj10060112

**Published:** 2022-06-15

**Authors:** Vitória Costa, António Sérgio Silva, Rosana Costa, Pedro Barreiros, Joana Mendes, José Manuel Mendes

**Affiliations:** 1 Department of Dental Sciences, University Institute of Health Sciences (IUCS), CESPU, 4585-116 Gandra, Portugal; vitoria_gcosta@hotmail.com (V.C.); rosana_gcosta@hotmail.com (R.C.); 2 UNIPRO—Oral Pathology and Rehabilitation Research Unit, University Institute of Health Sciences (IUCS), CESPU, 4585-116 Gandra, Portugal; pedro.barreiros@iucs.cespu.pt (P.B.); a27209@alunos.cespu.pt (J.M.); jose.mendes@iucs.cespu.pt (J.M.M.)

**Keywords:** precision, accuracy, dental implants, impressions, implant-supported prosthesis, computer-aided design, computer-aided manufacturing

## Abstract

With continuing technological developments, there have been advances in the field of fixed prosthetics, particularly in impression-taking techniques. These technological advances mean that a wide variety of diagnostic and/or rehabilitation possibilities can be explored without the need for physical models. The aim of this study was to evaluate the accuracy of three intraoral scanners used in oral implant rehabilitation using an extraoral scanner as a reference and varying the scanning area. Three models representing different clinical scenarios were scanned 15 times by each intraoral scanner and three times by the extraoral scanner. The readings were analyzed and overlaid using engineering software (Geomagic^®^ Control X software (Artec Europe, Luxembourg)). Statistically significant differences in accuracy were found between the three intraoral scanners, iTero^®^ (Align Technology Inc., San Jose, CA, USA), Medit^®^ (Medit^®^: Seoul, Korea), and Planmeca^®^ (Planmeca^®^: Helsinki, Finland). In all clinical scenarios, the iTero^®^ scanner had the best trueness (24.4 μm), followed by the Medit^®^ (26.4 μm) and Planmeca^®^ (42.1 μm). The Medit^®^ showed the best precision (18.00 μm) followed by the iTero^®^ (19.20 μm) and Planmeca^®^ (34.30 μm). We concluded that the iTero^®^ scanner had the highest reproducibility and accuracy in the clinical setting.

## 1. Introduction

Developments in digital technology and the recent introduction of the first intraoral scanner in dentistry have led to advances in the field of fixed prosthetics, particularly in impression-taking techniques [1,2]. This ongoing evolution has resulted in a wide variety of diagnostic and rehabilitation possibilities without the need to use physical models [3,4]. These devices have allowed us to digitize the oral cavity and create three-dimensional virtual models [4]. 

In the 1980s, a Swiss dentist, in collaboration with a Swiss electrical engineer, developed the first marketable impression-taking device (CEREC), which boosted the growth of computer-aided design (CAD) and computer-aided manufacturing (CAM) technology in dentistry [5,6]. This had the advantage of simplifying and improving previously complex and time-consuming techniques involved in the field of fixed prostheses during oral rehabilitation [7]. In dentistry, conventional impression-taking still requires scanning of a plaster model [6,8,9]. However, using CAD/CAM, it is possible to design a prosthesis and send the file directly to a milling machine [6]. The prosthesis produced is then placed and adjusted in the patient’s oral cavity by a dentist [6]. The advent of intraoral scanners means that it is now possible to acquire impressions directly without the need for conventional impression-taking [10]. The data obtained are electronically sent to the milling unit where the model is fabricated [6]. Thus, it has become possible to minimize errors arising from distortion, expansion, and contraction of the impression material [10]. Therefore, in the future, a dental prosthesis can be created with CAD software using a system known as standard tessellation language (STL), allowing a three-dimensional (3D) geometric description of the surface, without a representation of color, texture, or other attributes, to be scanned [11]. In this format, models can be evaluated either by overlaying images of the model (automatically by a computer using the best-fit algorithm) or by measuring two points in experimental and control groups using STL data [10,12,13,14,15]. One of the most important advances of this technology is the transition of files from closed to open [7]. Initially, the files were in a closed system, forcing scanning, drawing and milling to be performed with the same system. Nowadays, devices are increasingly adopting open systems, which allow more flexibility and freedom regarding the choice of the scanning and image processing systems in laboratories [16].

There are a number of other characteristics that differentiate the existing intraoral scanners [17,18,19]. Each device is based on different optical technology, such as active or passive triangulation, active wavefront sampling, confocal microscopy, and optical coherence tomography [6,20,21]. In general, all scanning systems combine more than one imaging technique to minimize inaccuracies that may arise during scanning, such as translucency and reflectivity of the target surface or relative motion, and to minimize humidity [6]. Clinically, a number of elements differentiate various devices, such as the ability to detect color impressions, tip size, and scanning speed [18,19,22]. The first generation of these devices requires the use of powder [19,22]. However, the more recent devices do not require opacification for impression-taking and are preferred because the use of powder can be inconvenient for patients [19,22]. The iTero^®^ Element Plus Series does not require opacification and has color-scanning features. The acquisition method for this device is based on parallel confocal microscopy [23,24]. Medit^®^ i500 scanners use the triangulation technique for 3D imaging and were introduced to the market to revolutionize 3D technology [25]. The image is based on a color video that enables the distinction between teeth, soft tissue, and tartar [25]. No powder is needed for scanning, which makes the procedure more comfortable for the patient. The data can be exported in several formats (STL/OBJ/PLY), which gives the operator some freedom of choice [25]. Based on the principles of confocal microscopy and optical coherence tomography, Planmecca^®^ Planscan use blue light with real-time color streaming video technology [22,26]. No opacification is required for scanning. An open system facilitates the conversion of acquired files into STL format, which is readable by all CAD systems [22]. 

When choosing an intraoral scanner, it is important to consider not only the ease of manipulation and image acquisition speed but also its accuracy [3,22,27]. Thus, the precision and trueness of the device should be carefully considered [27]. Trueness is the term used to describe the closeness between the mean value obtained from repeated measurements and the true value [17,22,28,29] and underpins all clinical applications involving implants or teeth [3]. On the other hand, precision refers to the closeness between independent measurements obtained under specific conditions [3,23,29]. These characteristics determine the accuracy of the device used (ISO 12836:2015) [3,22,23,28,30]. Ideally, intraoral scanners should be able to reproduce the surface of the scanned object as faithfully as possible (high trueness and precision) assigning consistent and repeatable results without any deviations [3]. Although several scanners with different characteristics, algorithms, and image acquisition methods are available in the market, few studies have addressed their accuracy, especially in implantology [3].

These scanners are powerful devices for the acquisition of optical impressions of dental structures and are replacing conventional impression-taking techniques in dentistry, which are unpleasant for some patients [3,4,19,31,32]. Unlike conventional methods, these impressions are more cost-effective, less time-consuming, more comfortable for patients, especially those with trismus or a strong gag reflex, can be viewed in virtual reality, are easy to transport, and allow better dynamic communication with the laboratory [4,8,19,22,32,33,34,35,36,37]. However, despite these advantages, intraoral scanners require an initial investment and that the clinician has the ability to ensure adequate scanning [34]. Studies have reported that inexperienced operators had worse scans than experienced users [38,39,40]. A more accurate scanning strategy minimizes inaccuracies in the digital fabrication workflow and yields accurate virtual 3D datasets [38,39,40]. 

Several methods have been developed to assess the precision and trueness of scanner devices. Some have either compared these devices to other intra-oral scanners or to traditional impression techniques [41,42]. A considerable number of researchers have either used an extraoral scanner or employed a master cast that has been measured by tactile computer metric measurements (CMM) to obtain reference data as a virtual 3D file [19,43]. The master model is then scanned by different intra-oral scanners, obtaining a virtual model [19,43]. These files are measured and compared to the reference date [19,43]. We have used the best-fit algorithm in our study. 

The primary aim of this study was to compare the accuracy and veracity of three intraoral scanners (iTero^®^ Element Plus Series, Medit^®^ i500, and Planmeca^®^ PlanScan) in oral implant rehabilitation using an extraoral scanner as a reference. A secondary aim was to determine whether the area scanned influences the accuracy of the data obtained by the scanner and whether the values obtained are within the reference range. The null hypothesis was that there would be no significant differences among the intraoral scanner devices and with the extraoral scanner. 

## 2. Materials and Methods

**Figure 1:**
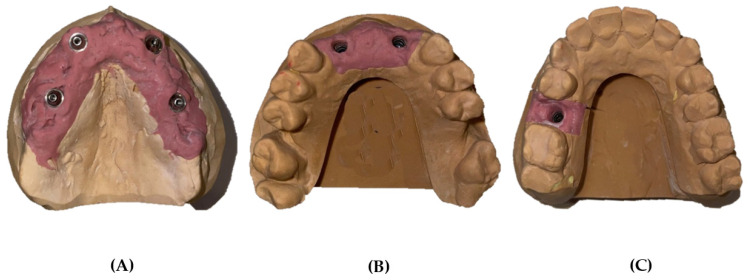
Three representative plaster models made in the laboratory. (**A**) Completely edentulous jaw rehabilitated with four implants. (**B**) Partially edentulous jaw rehabilitated with two implants. (**C**) Partially edentulous jaw rehabilitated with one implant.

The following three representative plaster models with artificial gingiva were made in the laboratory: model A, a total edentulous maxilla with four analog implants in positions 12, 15, 22, and 25 (Multi-unit/MegaGen^®^ analogs, MegaGen Implant Co., Ltd., Gyeongsan, Korea); model B, a partially edentulous maxilla with two analog implants in positions 12 and 22 (Analog/MegaGen AnyRidge); and model C, a partially edentulous maxilla with one analog implant in position 15 (Analog/MegaGen AnyRidge) (Figure 1).

Three ZrGEN-MegaGen AANISR4013, four ZrGEN-MegaGen, and AMUASR4013 scan bodies were used in the respective analogs to enable scanning and location of the implants. 

### 2.1. S6OO ARTI Extraoral Scanner

**Figure 2:**
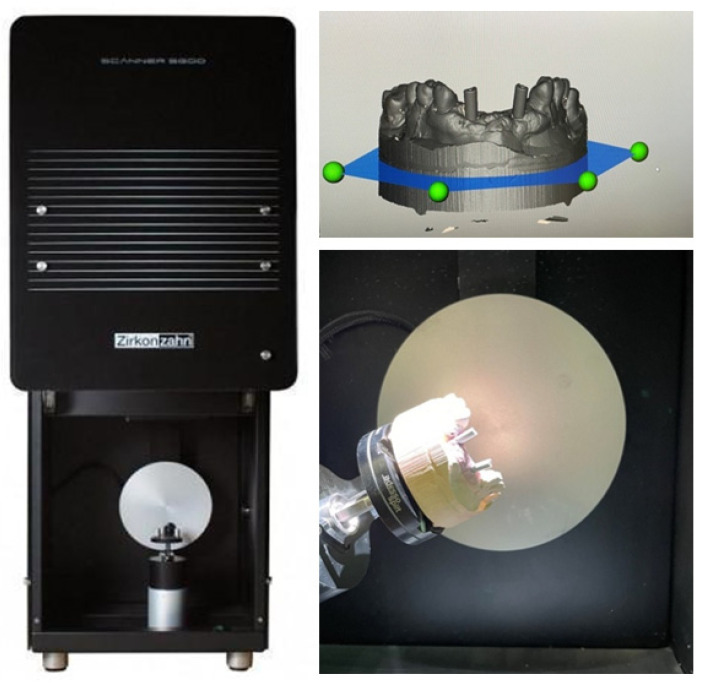
S600 ARTI extraoral scanner used in the laboratory.

We started by spraying the models with a white powder (Helling 3D; Willoughby, NSW, Australia) from approximately 20 cm away and then placing them inside the reading machine, where they were fixed to the rotating base that was moved so that the model could be read at various angles. This procedure was repeated three times for each model. The images obtained were named and saved in an STL file for subsequent analysis (Figure 2). Subsequently, the readings were entered into Geomagic^®^ Control X software (version 2018. 1.1; Artec Europe, Luxembourg), where the structures were superposed to select a reference dataset. These models were used as a guide for measurement of the veracity of all intraoral scanners.

### 2.2. Intraoral Scanners

**Figure 3:**
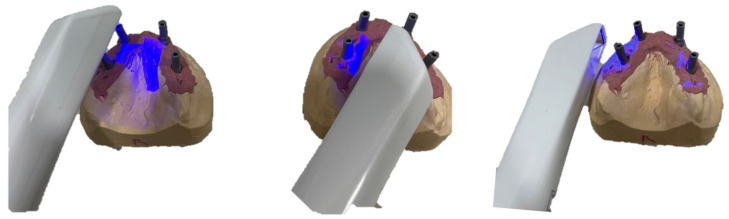
Scanning technique used by intraoral scanners.

The three intraoral scanners evaluated were the iTero^®^ Element Plus Series (Align Technology Inc., San Jose, CA, USA), Medit i500 (Medit^®^; Seoul, Korea), and Planmeca^®^ PlanScan (Planmeca^®^: Helsinki, Finland). To evaluate the accuracy of these devices, the models were scanned 15 times per scanner with a 10-min interval to allow for cooling, resulting in a total of 135 virtual 3D models. (Table 1 and Figure 3). All measurements were acquired by the same operator to reduce the risk of bias and ensure that the same environmental conditions were maintained, in a room with a temperature of 22 °C. The calibration of all intraoral scanners was performed according to the manufacturer’s recommendations. 

#### 2.2.1. iTero^®^ Element Plus Series

The iTero^®^ Element Plus Series is a device that does not require opacification and features color scanning. The acquisition method of this device is based on parallel confocal microscopy. The scanning procedure with iTero^®^ started from the oclusal surface, rolling to palatal and buccal surface.

#### 2.2.2. Medit^®^ i500

Using the triangulation technique to acquire 3D images, MEDIT^®^ was introduced in the market in order to improve and revolutionize 3D technology. The image is based on a color video enabling the distinction between teeth, soft tissue and tartar. It does not require the use of powder for scanning, which makes the procedure more comfortable for the patient. This allows data to be exported in several formats (STL/OBJ/PLY), giving the operator freedom of choice. The scanning strategy for the Medit group was performed by zigzag movement, from oclusal to palatal and buccal surface.

#### 2.2.3. Planmeca^®^ PlanScan

Based on the principle of Confocal Microscopy and Optical Coherence Tomography, this system uses a blue light with real-time and color streaming video. No opacification is required for scanning. This open system facilitates the conversion of acquired files into STL readable by all CAD systems. The scanning technique from Planmecca, Planscan started first from the oclusal, rotating to the palatal and then rotating across the distal proximal to reach the buccal side.

### 2.3. Alignment and Measurement Procedures

**Figure 4:**
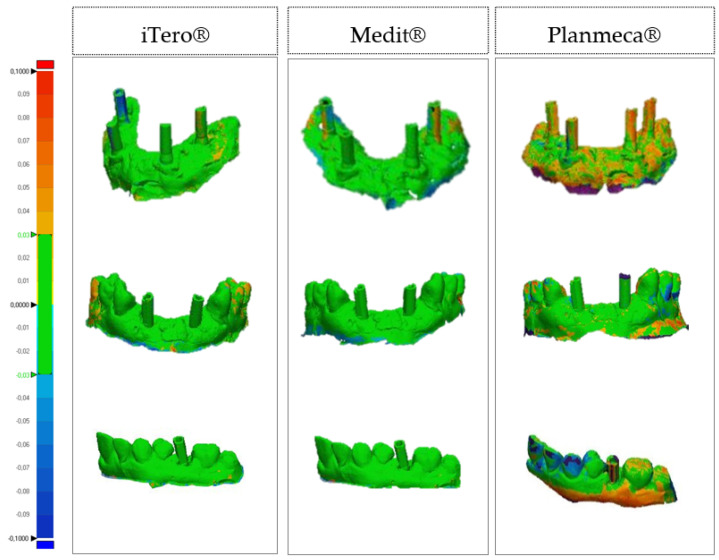
Colorimetric maps comparing the trueness of three intraoral scanning.

**Figure 5:**
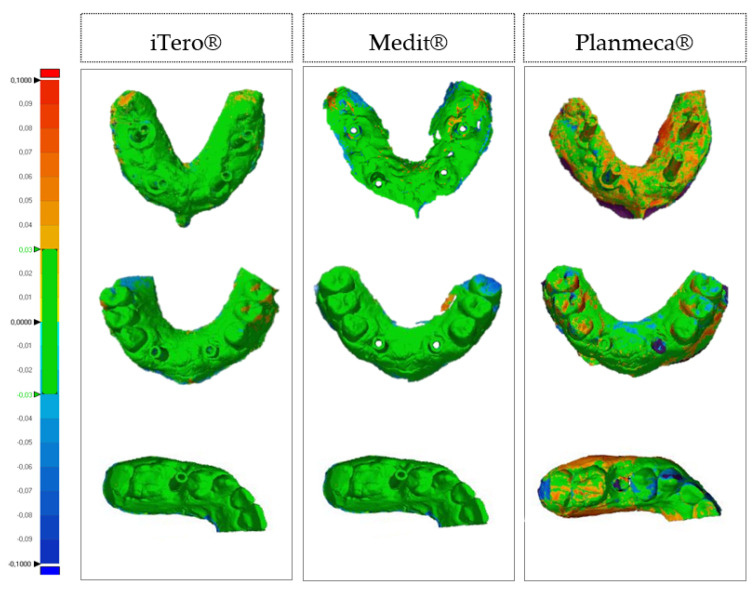
Colorimetric maps comparing the precision of three intraoral scanning models.

After gathering 15 impressions per scanner (iTero^®^, Medit^®^, and Planmeca^®^), the 3D images were transformed into STL format files and then manipulated digitally using the 3D analysis program Geomagic Control X version 2018. Once imported into the software, all the obtained images were cut according to planes to delimit only the area of interest. The images were then superimposed two by two according to the best-fit algorithm to assess trueness (superimposition of laboratory reference and intraoral scans) and precision (superimposition of the scan with the best trueness on the different intraoral scanners used). Next, using the “3D Deviation” function, the distance between specific points was quantified. The color maps indicated the displacements between overlapped structures. The same colorimetric parameters were set for the different models; the maximum deviation ranged from 100 μm to–100 μm, with the best results ranging between 30 μm and −30 μm (green; Figure 4 and Figure 5). After completion of the analysis of all overlays, the numeric data obtained were exported to a report. 

### 2.4. Datasets and Statistical Analysis

Fifteen trueness measurements were evaluated for the iTero^®^, Medit^®^ and Planmeca^®^ scanners and for models A, B and C, for a total of 135 measurements. To evaluate the precision, 14 measurements per scanner and model were included for a total of 126 measurements. Discrepancies between the reference measurements and the measurements obtained by the scanners were calculated using the root mean square (RMS). This is often used in cases in which there is an evaluation system with positive and negative discrepancies, thus, preventing deviations from canceling out when they are summarized by averaging or summation. Thus, the sum of the RMS for all deviations between the point evaluated by the scanner and the reference point was calculated.

$$\mathrm{RMS}=\frac{1}{\sqrt{n}}\sqrt{\sum {\left(x_{1,i}-x_{2,i}\right)}^{2}}$$

Point *x*(1,*i*) refers to the point on the scanner being tested and point *x*(2,*i*) refers to the same point on the reference model. The squared sum of the differences reflects the amount of error. The square root places this value on the scale of the original error distance. Dividing by the number of observations reflects the average error between the reference model and the scanners tested.

The RMS trueness and precision values were compared between the iTero^®^, Medit^®^, and Planmeca^®^ scanners and models A, B, and C using two-way analysis of variance. The effect size was calculated using the eta2 (η^2^) value, considering cut-off points of 0.01 as a mild effect, 0.06 as a moderate effect, and 0.14 as a high effect.

The Student’s one-sample *t*-test was used to evaluate the statistical significance and magnitude of the mean difference evaluated by RMS with respect to “zero”, which means total overlap between the reference model and the tested scanner. The effect size “d” was evaluated according to cut-off points of 0.20 for a slight effect, 0.50 for a moderate effect, and 0.80 for a high effect. The RMS values across the three scanners and three models are summarized as the mean and standard deviation. All data were analyzed using SPSS software version 22 (IBM Corp., Armonk, NY, USA). The significance level set for rejection of the null hypothesis was 5%.

## 3. Results

Table 2 show the RMS values obtained for trueness and precision by the different scanners and models using the Geomagic Control X software. Statistically significant differences in veracity values were observed, namely, F_(2,126)_ = 673.53, *p* < 0.001, η^2^ = 0.92, as well as in the comparison per model [F(_2,126)_ = 58.13, *p* < 0.001, η^2^ = 0.48] and the interaction between them [F_(4,126)_ = 17.77, *p* < 0.001, η^2^ = 0.36]. 

This table shows that the iTero^®^ scanner achieved the best results in an edentulous jaw rehabilitated with four implants (model A), an edentulous jaw partially rehabilitated with two implants (model B). and an edentulous jaw partially rehabilitated with one implant (model C) (24.40, 24.40, and 24.90 μm respectively). The next best veracity results were obtained by the Medit^®^ (model C, 26.4 μm; model B, 32.90 μm; model A, 37.90 μm) followed by the Planmeca^®^ (model C, 42.10 μm; model B, 46.90 μm; model A, 50.7 μm). 

Table 3 shows the differences between the RMS value for trueness obtained for each scanner and model and the “zero” point, which indicates total overlap between the reference model and the scanners used. All tests were statistically significant (*p* < 0.001) with high effect sizes, suggesting a significant departure from the zero point of total overlap. Table 3 suggests that the RMS distribution for the trueness of the scanner evaluations was higher, particularly for the iTero^®^ in model A and model C and for the Planmeca^®^ in model A, model B, and model C.

Table 4 shows the results of the comparison of RMD values for precision by scanner type and model. Statistically significant differences were observed in the comparison by scanner [F(_2,117)_ = 593.52, *p* < 0.001, η^2^ = 0.91], comparison by model [F_(2,117)_ = 218.95, *p* < 0.001, η^2^ = 0.79], and in the interaction between scanner and model, [F(_4,117)_ = 24.01, *p* < 0.001, η^2^ = 0.45].

Tukey’s multiple comparisons tests identified differences in the RMS between scanners (*p* < 0.001) and between models (*p* < 0.001). Table 4 shows that the iTero^®^ scanner obtained the best precision in model B and model C. The Medit^®^ scanner had the lowest RMS value for precision in the model C whereas the Planmeca^®^ scanner had the highest RMS value for precision. The model C values for iTero^®^ and Medit^®^ were significantly higher than those obtained in the other models (*p* < 0.001).

Table 5 shows the differences between the RMS values for precision obtained for each scanner and model and the “zero” point that indicates total overlap between the reference model and the scanners used. All tests were statistically significant (*p* < 0.001) with high effect sizes, suggesting a significant departure from the “zero” point of total overlap. The data in Table 5 suggest that the RMS values for the precision of the scanner evaluations were high, particularly for the iTero^®^ (model B), Medit^®^ (model C) and Planmeca^®^ (model B) scanners.

## 4. Discussion

With advances in technology and the introduction of a wide range of machines, devices, and software, a digital revolution has taken place worldwide, and dental medicine is no exception [19,22]. Currently, through image acquisition systems such as cone-beam computed tomography, intraoral scanners, and facial scanners, transition from the real world to the virtual world can be easily achieved. Sometimes, the absence of reference points, characteristics of the surface to be scanned, scanning strategy, sensor size and software, among other factors, may affect the accuracy of the impression [16,19,22,44,45,46,47,48,49,50]. This step is important because inaccuracies may cause stresses and mismatches in the final work [50].

In 2012, Van der Meer et al. were the first to compare the accuracy of intraoral scanners (iTero, Lava COS^®^, CEREC AC, Bluecam^®^) in implantology [19]. They used plaster models with partial rehabilitation using three implants and polyether ether ketone screwable scan bodies and took readings using an industrial scanner to obtain a reference model [12]. With these readings, after using the intraoral scanners, the scans were imported into 3D model overlay software, and the angulation and distance between the markers were evaluated and compared with the reference models [12]. The Lava COS scanner is the device with the most consistently low error [12]. In that study, the authors concluded that with increasing distance, angulation errors could be expected along the arch because of accumulation of registration errors during the scanner’s advancement in space [12]. Similarly, a study by Mangano et al. in 2019 found that the error increased from partial rehabilitation to full rehabilitation [3]. In that study, the critical trueness threshold was set at 30 μm [3], and there are authors who consider discrepancies >30 μm to be acceptable and those that are <150 μm as the limit to avoid long-term complications [41]. Applying the established 30 μm of trueness, we identified some devices in the literature that achieved good results [3,51,52,53,54]. Many studies have reported the clinical value of the trueness and precision of intraoral scanners both in vivo and in vitro [3,51,52,53,54].

Mangano et al. established a mean accuracy value of 30 μm [3], whereas Ender et al. determined a mean precision value of 4–16 μm and a mean trueness value of 20–48 μm for partial impressions when comparing them with the values for conventional impressions [47]. Current intraoral devices are clinically adapted to common practice with an accuracy that is at least similar to that of conventional impressions [17,18]. Nevertheless, Mϋhlemann et al. reported a higher precision in plaster models (32 ± 11 μm) in their in vitro research using the conventional technique when compared with the precision in 3D models obtained with digital impressions (57 ± 32 to 176 ± 120 μm) [55]. Operator calibration and the fact that these were milled models may have contributed to the relatively poor accuracy for intraoral scanners [55]. Koch et al. reported that software and scanner errors caused changes in their milled models [56].

Some studies have found conventional impressions to be superior to digital impressions, with deviations in the range of 26–56 ± 29 μm and 15.6–176.7 μm [14,55,57,58,59,60,61,62,63,64,65,66,67].

In our in vitro study, three plaster models with artificial gingiva were prepared with four, two, and one implants that had Zr GEN-MegaGen scan bodies threaded onto them. These models were scanned using an extraoral scanner (S600; Zirkonzahn, Gais, Italy), which was used as a reference, and three intraoral scanners (iTero^®^, Medit^®^, and Planmeca^®^). For each model, 15 readings were obtained using the different devices. Geomagic Control X engineering software was used to overlay the reference model to calculate the trueness and between each scanning to measure the precision. With regard to trueness, a rather small deviation from the reference model was noted for partial rehabilitation with an implant. iTero^®^ had the best trueness (24.90 μm) but Medit^®^ also had values below the critical threshold set at 30 μm (26.40 μm). 

Although the Planmeca^®^ has a mean value >30 μm with a trueness of 42.10 μm, its deviation from the reference model was among the lowest. That is, the variation within each group was very small, confirming the high reliability and repeatability of the results when scanning an implant (Table 2). However, statistically significant differences were found between the different scanners. When scanning two implants for a 4-unit bridge, iTero^®^ remained below the critical value, achieving its best result (24.40 μm) and Medit^®^ showed slightly higher averages (32.90 μm). These results (Medit^®^) are consistent with those of other studies that reported a progressive increase in intraoral scanner error with an increase in the area scanned [3,12,14,59]. The iTero^®^ and Medit^®^ had excellent results, compatible with their successful clinical use in similar clinical situations. This is in line with the current literature [14,46,48,59]. We obtained statistically significant differences in precision between the different scanners and models. For oral rehabilitation with one implant, Medit^®^ had the lowest precision at 18.00 μm, followed by the iTero^®^ (19.2 μm) and Planmeca^®^ (34.3 μm). With the two implants, the precision results were higher than previous results, as expected. Despite the slight difference between the values of the iTero^®^ (25.00 μm) and Medit^®^ (26.80 μm) scanners, the Planmeca^®^ scanner had a noticeably higher value of 53.00 μm, the Planmeca^®^ still shows the most constant deviations (Table 4). 

In the last 5 years, the precision of multi-unit impressions has received increasing attention [6,23,41,53,54,60,61,62,63,64,65,66,67]. For total rehabilitations on multi-units, some authors have reported errors >100 μm [22,38,61,62,63], while others have reported lower values [12,53,64]. The different results can be explained by the different methodologies used by the scanners [12] and by the presence or absence of the teeth remaining between the implants serving as reference points [61]. Some studies using new-generation intraoral scanners (Cerec Omnicam^®^, CS3500^®^, Color^®^, TRIOS^®^, True Definition^®^, Emerland^®^) have reported deviations <100 μm [3,22,39,52,63,65]. All have used models of total rehabilitation with four or six implants. Two studies that used the Planmeca^®^ PlanScan and True Definition found slightly higher values of 253.4 ± 13.6 μm and 106.4 ± 23.1 μm, respectively [19,22].

In our study, the trueness values were lower (iTero^®^, 24.40 μm) in representative situations of fully edentulous patients rehabilitated with four implants than those in single implant rehabilitations (iTero^®^, 24.90 μm). Contrary to what some authors have reported, they found an increase in error with an increase in the area scanned [2,12,14,59]. Medit^®^ was the device with the second-best trueness value (37.90 μm) followed by Planmeca^®^ at 50.70 μm. Our findings are in line with those of other authors, who mentioned deviations of <100 μm as a reference [3,22,39,52,65]. Statistically significant differences in the precision were found between multiple devices and models. Again, the iTero^®^ was found to be the most accurate (26.00 μm), followed by the Medit^®^ (35.90 μm) and Planmeca^®^ (57.30 μm).

Given these values, the best result was achieved by iTero^®^, which confirmed a pattern of high stability when comparing the quality of different readings. However, the data resulting from acquisition with various scanners could hypothetically be used to generate and produce successful implant restorations in full arches. These findings are important because until the recent use of first-generation scanners in similar clinical situations, it was impossible or difficult to achieve such accuracy [13,22,47,68].

The generation and type of intraoral scanner seem to influence the scanning precision, with some devices showing better precision in full scans than others [35,43,69,70]. Nevertheless, Mangano et al. found that the accuracy of digital impressions was not correlated with the resolution of the device in fully edentulous patients [3]. Although some studies have found that digital printing is better than conventional impressions [66,67]. In this study, a new generation of intraoral scanners (CEREC Omincam^®^, True definition^®^ and Trios 3Shape^®^) was used, with deviations <46 μm [66,67]. Others have found that not all scanners can be used for edentulous patients [71]. According to Miyoshi et al., the extent of the impression influences the accuracy of the scan [72]. Digital impressions should be limited to smaller prostheses with three-element frameworks supported by two implants [72]. However, researchers have established that digital printing is less precise than traditional printing [73,74].

In vivo, the effectiveness of these devices seems to be compromised by several factors, including the presence of saliva, object movements, and limitations of the device or operator [64]. The area to be scanned is also an important factor, because more distant areas in an everyday clinical situation may be more difficult to reach [64]. Missing gums, cheek mobility, and the tongue also contribute negatively to the quality of the scan [64]. 

Although the number of studies on the accuracy of different scanners has increased, there is still a need for more scientific evidence in implantology [3,12,22,59,75].

## 5. Conclusions

Based on the data obtained and considering the limitations of this in vitro study, we conclude that the iTero^®^ is the best intraoral scanner, which confirmed a high stability pattern in our comparison of the quality of the different readings randomized to specific clinical situations. Trueness was slightly better for total rehabilitation than for partial rehabilitation (iTero^®^), reflecting the great progress made by the latest generation of intraoral scanners. 

## Figures and Tables

**Table 1:** Information about the intraoral scanner systems.

System	Manufacturer	Scanning Technology	Scan Protocol	Acquisition	Powder Application	Export
iTero- Element Plus Series	Align Technology	Parallel confocal microscopy	OPB	Video Sequence	No	STL/OBJ/PLY
i500	Medit	Triangulation technique	OPB	Video Sequence	No	STL/OBJ/PLY
Planscan	Planmecca	Confocal microscopy and optical coherence tomography	OPB	Video Sequence	No	STL/OBJ/PLY

O = Occlusal; P = Palatal; B = Bucal.

**Table 2:** Comparison of root mean square values for trueness according to type and model of scanner by two-way analysis of variance.

				Two-Way Analysis of Variance		
	Model A	Model B	Model C	Scanner	Model	Interaction
iTero^®^	0.0244 (0.0017)	0.0244 (0.0047)	0.0249 (0.0012)	F_(2,126)_ = 675.53 *p* < 0.001 η^2^ = 0.92	F_(2,126)_ = 58.13 *p* < 0.001 η^2^ = 0.48	F_(4,126)_ = 17.77 *p* < 0.001 η^2^ = 0.36
Medit^®^	0.0379 (0.0028)	0.0329 (0.0041)	0.0264 (0.0030)			
Planmeca^®^	0.0507 (0.0028)	0.0469 (0.0017)	0.0421 (0.0019)			

Data are presented as the mean and standard deviation in millimeters. *p* < 0.001, statistically significant difference between scanners and between brands, Tukey’s test.

**Table 3:** Comparison of the root mean square values for trueness in relation to the “zero” error.

	*t*-Test, H0: μ = 0		
	Model A	Model B	Model C
iTero^®^	t_(14)_ = 56.92 (*p* < 0.001) d = 14.70	t_(14)_ = 20.22 (*p* < 0.001) d = 5.22	t_(14)_ = 80.19 (*p* < 0.001) d = 20.71
Medit^®^	t_(14)_ = 52.91 (*p* < 0.001) d = 13.66	t_(14)_ = 30.89 (*p* < 0.001) d = 7.98	t_(14)_ = 34.34 (*p* < 0.001) d = 8.87
Planmeca^®^	t_(14)_ = 69.32 (*p* < 0.001) d = 17.90	t_(14)_ = 108.10 (*p* < 0.001) d = 27.91	t_(14)_ = 84.03 (*p* < 0.001) d = 21.70

Comparisons were made using the *t*-test.

**Table 4:** Comparison of root mean square values for precision according to type and model of scanner by two-way analysis of variance.

				Two-Way Analysis of Variance		
	Model A	Model B	Model C	Scanner	Model	Interaction
iTero^®^	0.0260 (0.0039)	0.0250 (0.0025)	0.0192 (0.0042)	F_(2,117)_ = 593.52 *p* < 0.001 η^2^ = 0.91	F_(2,117)_ = 218.95 *p* < 0.001 η^2^ = 0.79	F_(4,117)_ = 24.01 *p* < 0.001 η^2^ = 0.45
Medit^®^	0.0359 (0.0052)	0.0268 (0.0052)	0.0180 (0.0020)			
Planmeca^®^	0.0573 (0.0034)	0.0530 (0.0018)	0.0343 (0.0027)			

Data are presented as the mean and standard deviation in millimeters. *p* < 0.001, statistically significant difference between scanners and between brands, Tukey’s test.

**Table 5:** Comparison of root mean square values for precision against the “zero” error.

	*t*-Test, H0: μ = 0		
	Model A	Model B	Model C
iTero^®^	t_(13)_ = 24.76 (*p* < 0.001) d = 6.62	t_(13)_ = 36.84 (*p* < 0.001) d = 9.85	t_(13)_ = 17.28 (*p* < 0.001) d = 4.62
Medit^®^	t_(13)_ = 25.86 (*p* < 0.001) d = 6.91	t_(13)_ = 21.11 (*p* < 0.001) d = 5.64	t_(13)_ = 32.90 (*p* < 0.001) d = 8.79
Planmeca^®^	t_(13)_ = 63.08 (*p* < 0.001) d = 16.86	t_(13)_ = 110.81 (*p* < 0.001) d = 29.62	t_(13)_ = 48.52 (*p* < 0.001) d = 12.97

Comparisons were made using the *t*-test.

## Data Availability

The data that support the findings of this study are available from the corresponding author upon request.
